# qPCR assay for detection of Woodchuck Hepatitis Virus Post-Transcriptional Regulatory Elements from CAR-T and TCR-T cells in fresh and formalin-fixed tissue

**DOI:** 10.1371/journal.pone.0303057

**Published:** 2024-06-06

**Authors:** Shalini Pullarkat, Graeme Black, Marie Bleakley, Denise Buenrostro, Aude G. Chapuis, Alexandre V. Hirayama, Carla A. Jaeger-Ruckstuhl, Erik L. Kimble, Bo M. Lee, David G. Maloney, Jerald Radich, Brandon W. Seaton, Jennifer M. Specht, Cameron J. Turtle, David W. Woolston, Jocelyn H. Wright, Cecilia C. S. Yeung

**Affiliations:** 1 Translational Science and Therapeutics Division, Fred Hutchinson Cancer Center, Seattle, Washington, United States of America; 2 Clinical Research Division, Fred Hutchinson Cancer Center, Seattle, Washington, United States of America; 3 Department of Pediatrics, University of Washington, Seattle, Washington, United States of America; 4 Program in Immunology, University of Washington, Seattle, Washington, United States of America; 5 Department of Medicine, Division of Hematology and Oncology, University of Washington, Seattle, Washington, United States of America; 6 Integrated Immunotherapy Research Center, Fred Hutchinson Cancer Center, Seattle, WA, United States of America; 7 Department of Laboratory Medicine and Pathology, University of Washington School of Medicine, Seattle, Washington, United States of America; Pennsylvania State University Hershey Medical Center, UNITED STATES

## Abstract

As adoptive cellular therapies become more commonplace in cancer care, there is a growing need to monitor site-specific localization of engineered cells—such as chimeric antigen receptor T (CAR-T) cells and T-cell receptor T (TCR-T) cells—in patients’ tissues to understand treatment effectiveness as well as associated adverse events. Manufacturing CAR-T and TCR-T cells involves transduction with viral vectors commonly containing the *WPRE* gene sequence to enhance gene expression, providing a viable assay target unique to these engineered cells. Quantitative PCR (qPCR) is currently used clinically in fresh patient tissue samples and blood with target sequences specific to each immunotherapy product. Herein, we developed a *WPRE*-targeted qPCR assay that is broadly applicable for detection of engineered cell products in both fresh and archival formalin-fixed paraffin embedded (FFPE) tissues. Using both traditional PCR and SYBR Green PCR protocols, we demonstrate the use of this *WPRE*-targeted assay to successfully detect two CAR-T cell and two TCR-T cell products in FFPE tissue. Standard curve analysis reported a reproducible limit of detection at 100 *WPRE* copies per 20μL PCR reaction. This novel and inexpensive technique could provide better understanding of tissue abundance of engineered therapeutic T cells in both tumor and second-site toxicity tissues and provide quantitative assessment of immune effector cell trafficking in archival tissue.

## Introduction

Chimeric antigen receptor T cell (CAR-T cell) and engineered T-cell receptor T cell (TCR-T cell) therapy are adoptive cellular therapies developed by modifying T cells to target specific cell surface antigens and peptides presented in the context of human leukocyte antigens (HLA), respectively [1]. These immunotherapies allow for targeted treatment approaches for relapsed or refractory hematologic malignancies as well as solid tumors [2–5]. Lentiviral vectors used to engineer targeted cells for these therapies contain enhancer sequences that promote mRNA stability to increase gene expression [6]. The Woodchuck Hepatitis Virus Posttranscriptional Regulatory Element (*WPRE*) is a transcription-enhancing gene sequence commonly used to enhance the expression of transgenes delivered by retroviral and lentiviral vectors [7–9]. This gene sequence is a unique element employed in the manufacturing of immune effector cells and does not naturally occur in the human genome.

As adoptive cellular therapies grow more commonplace in cancer treatment, downstream adverse events (AEs) and safety monitoring questions grow more relevant. Unique AEs associated with *in vivo* expansion of CAR-T and TCR-T cells include cytokine release syndrome (CRS) and immune effector cell-associated neurotoxicity syndrome (ICANS)—both of which can be life-threatening [10–14]. CRS can be associated with circulatory collapse and multiorgan injury, such as pulmonary, renal, hepatic, and cardiac dysfunction [15]. Additionally, current literature report potentially severe GI-related AEs in 28% of patients treated with CAR-T cell therapy [16]. Adverse cardiovascular and pulmonary events (such as tachyarrhythmias, cardiomyopathy, pericardial and pleural disorders, venous thromboembolism, and myocarditis) have also been reported, and have been fatal [17,18]. These complications have long been believed to be mainly cytokine mediated. However, the role of engineered T cells in the pathophysiology of these types of AEs is not fully understood due to insufficiency of current methods for detecting immunotherapy cells in tissue. Additionally, knowledge of the trafficking kinetics of engineered cells to target tumor site provides valuable insight for translational research understanding the homing and persistence of CAR-T/TCR-T within the body and enhance the development of comprehensive methods and products.

Herein, we developed and optimized a quantitative PCR (qPCR) assay to detect the *WPRE* sequence for use on human biopsy and resected tissues, including fresh and formalin-fixed, paraffin embedded (FFPE) samples to monitor immune effector cell infiltration. We demonstrate assay effectiveness using DNA at a variety of concentrations from three different CAR-T cell and TCR-T cell products in both fresh and FFPE human tissue samples. With this simple assay, we can detect genetically modified T cells, regardless of product type, based on the presence of the *WPRE* gene sequence.

## Materials and methods

### Sample acquisition

The development of this assay utilized one CAR-T cell product and two TCR-T cell products: JCAR014 anti-CD19 CAR-T cells, MAGEA1-targeted TCR-T cells, and HA-1-targeted TCR-T cells (CD4- and CD8-positive) [19–21]. Pre-infusion cell products and CAR-T and TCR-T cell plasmids were obtained from the laboratory which developed the engineered cell products; JCAR014 CAR-T cells from the Turtle Lab, MAGEA1-targeted TCR-T cells from the Chapuis Lab, and HA-1-targeted TCR-T cells from the Bleakley Lab, all of Fred Hutchinson Cancer Center’s (FHCC) Translation Science and Therapeutics Division. These samples were split into fresh frozen pellets and FFPE-processed cell blocks in admixtures with human donor peripheral blood mononuclear cells (PBMCs) to mimic FFPE tissues. DNA extracted from fresh or frozen CAR-T cells, TCR-T cells, and PBMCs from healthy donors, as well as DNA extracted from FFPE PBMC pellets and FFPE CAR-T/TCR-T cell and PBMC pellet admixtures were used for assay development.

During primer selection and protocol development using traditional PCR, pre-infusion CAR-T and TCR-T cell products were tested alongside an ML1 cell line transduced to express green fluorescent protein using a *WPRE*-containing lentiviral vector (Table 1). DNA obtained from unmodified PBMCs and the K-562 cell line (ATCC) were used as negative controls. Human tonsil tissue FFPE samples were added as negative control tissues to further optimize this assay in qPCR.

**Table 1:** List of samples used for assay development and validation.

Sample	Sample use	Sample Type	Diagnosis	CAR-T/TCR-T cell target	Tissue Source
Healthy donor PBMC** **	Negative control	Fresh, FFPE	N/A	N/A	Cell pellet
K-562 cell line** **	Negative control	Fresh, FFPE	N/A	N/A	Cell pellet
ML1 GFP cell line** **	Positive control	Fresh	N/A	N/A	Cell pellet
JCAR014 CAR-T cells	Positive control	Fresh, FFPE ^a^, Plasmid	N/A	CD19	Cell pellet, plasmid DNA
CD4+ HA-1 TCR-T cells	Positive control	Fresh, FFPE ^a^	N/A	HA-1	Cell pellet
MAGEA1 TCR-T	Positive control	Fresh, FFPE ^a^, Plasmid	N/A	MAGEA1	Cell pellet, plasmid DNA
Patient 1	Infused	FFPE	DLBCL ^b^	CD19	Excision biopsy
Patient 2** **	Infused	FFPE	TNBC—Metastatic	ROR1	Needle Biopsy
Patient 3** **	Infused	FFPE	TNBC—Metastatic	ROR1	Needle Biopsy
Patient 4** **	Infused	FFPE	TNBC—Metastatic	ROR1	Needle Biopsy
Patient 5** **	Infused	FFPE	TNBC—Metastatic	ROR1	Needle Biopsy
Patient 6** **	Infused	FFPE	CLL ^d^	ROR1	Decalcified Marrow Core Biopsy
Patient 7** **	Infused	FFPE	NSCLC^e^	ROR1	Needle Biopsy
Patient 8** **	Infused	FFPE	NSCLC^e^ - Metastatic	ROR1	Needle Biopsy
Patient 9** **	Infused	FFPE	TNBC	ROR1	Needle Biopsy
Healthy donor tonsil	Negative control	FFPE	N/A	None	Excision Biopsy

^a^ FFPE cell pellet admixture.

^b^ DLBCL–Diffuse large B-cell lymphoma.

^c^ TNBC–Triple negative breast cancer.

^d^ CLL–Chronic lymphocytic leukemia.

^e^ NSCLC–Non-small cell lung cancer.

Patient samples were obtained under FHCC protocol 8757, allowing for use of previously collected research and clinical tissues for the development of molecular and translational assays. This study received IRB approval by the University of Washington Human Subjects Division (IRB ID: STUDY00014352), including a waiver of consent. Patient records were accessed on May 5, 2023 and September 18, 2023 to obtain information regarding initial diagnoses. Patient samples were then de-identified.

### DNA extraction

Genomic DNA from fresh cells was extracted with the QIAGEN Gentra Puregene Blood kit (QIAGEN INC, Hilden, Germany. Cat# 158023) according to manufacturer’s instructions. DNA concentrations were verified using the Qubit 1x dsDNA High Sensitivity assay (Thermo Fisher Scientific Inc, Carlsbad, CA, USA. Cat# Q32851). Genomic DNA extraction from FFPE tissue samples was performed using the QIAamp DNA FFPE Tissue Kit (QIAGEN INC, Hilden, Germany. Cat# 56404) according to manufacturer instructions, with one deviation whereby samples received two washes of AW1 and AW2 buffers, in sequence, to ensure the cleanest possible product. From FFPE tissues, DNA extractions were performed on 15 μm curl sections, and unstained slides of 4 μm sections, cut from the FFPE block. For cell pellets and tissue for patient 1, two 15 μm curls were cut for DNA extraction. For patients 2–9, tissue was removed from three unstained slides each containing a 4 μm section of tissue. These FFPE patient samples were deparaffinized by using xylene and ethanol washes, and DNA was extracted using the QIAamp DNA FFPE Tissue Kit. The final eluate volume was 20–30μL to maximize eluted DNA concentration.

### PCR primers and protocol development

Forward and reverse primers were designed using NCBI Primer Blast to design a pair specific to the *WPRE* region and purchased from IDT (Integrated DNA Technologies, San Diego, CA). Multiple candidate primer combinations were tested and cross-matched for the selection of the most specific and consistent primer pair. The chosen primer pair generated an amplicon of 159 base pairs (bp) (Forward: 5’ – GTG GAT ACG CTG CTT TAA TGC CT – 3’; Reverse: 5’ – GTT GCG TCA GCA AAC ACA GT – 3’). We tested the specificity and sensitivity of our primer pair combinations with a standard PCR protocol (annealing temperature 67°C) and ran these results through gel electrophoresis, to verify the correct product size. These primers were also run on a temperature gradient to find optimal conditions for PCR cycling to avoid spurious background amplification on the Bio-Rad C1000 Touch Thermal Cycler. The selected primers and thermocycling protocol were then adapted for use with the Applied Biosystems (ABI) PowerTrack^TM^ SYBR Green MasterMix (Thermo Fisher Scientific Inc., Waltham, MA. Cat# A46012) as follows: 95°C for 2 minutes, [(95°C for 15 seconds, 60°C for 1 minute) x 40 cycles], melt curve from 65°C to 95°C, increment 0.5°C for 5 seconds. The primers were used at a concentration of 8 μM, and an additional melt curve step was added for result analysis. PCR was run on the Bio-Rad CFX 384 Real Time PCR Detection System and the ABI QuantStudio^TM^ Real-Time PCR system to test for consistency between instruments.

### PCR product verification and assay controls

The detection of *WPRE* DNA distinct from non-specific background or other viral product amplification was confirmed by various methods. In general, correctly sized PCR products were verified for all positive samples by 2% agarose gel electrophoresis. For qPCR, melt curve analysis was routinely performed to ensure that the reported amplicon had the expected T_m_. Product T_m_ peaks seen between 81.00–84.50°C were accepted as positive. All other T_m_ were interpreted as negative results.

Admixtures of DNA extracted from negative control FFPE tonsil tissue and CAR-T/TCR-T cell plasmid DNA were created to mimic the conditions of an FFPE patient sample. These “spiked” samples were made from a 1:100 dilution by volume of plasmid DNA at 10^5^ copies of *WPRE* DNA, in a background of DNA extracted from FFPE tonsil, and served as run controls during the product verification stages of assay development.

### Constructing and using standard curves for copy number analysis

JCAR014 CAR-T cell and MAGEA1 TCR-T cell plasmid DNA were serially diluted 10-fold to generate standards with a *WPRE* copy number from 10^8^ copies to 10 copies per 20μL PCR reaction. This allowed for the construction of DNA standard curves for the two products to accompany clinical samples. In stability experiments for these standards, the standard curve controls created with plasmid DNA diluted in TE buffer with 1 ng/μL of human tonsil DNA, when stored at -80°C in single use aliquots, yielded the most consistent results.

**Fig 1:**
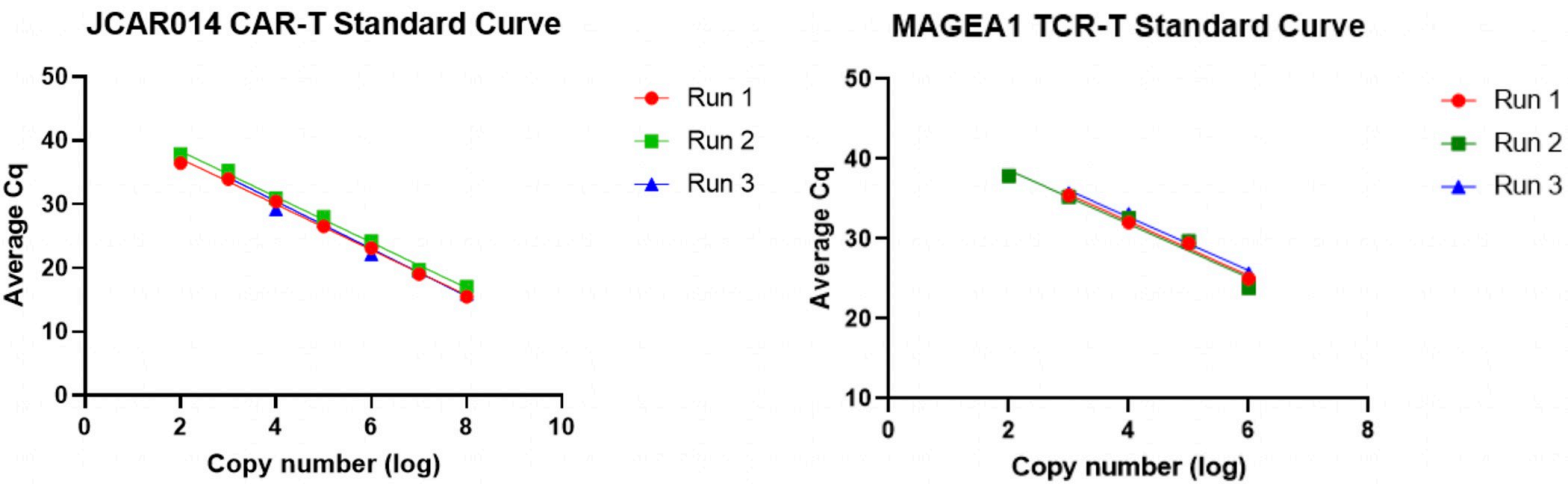
Plasmid standard curves. (A) JCAR014 CAR-T cell plasmid standard curves using data points from three different PCR runs, with reactions run in triplicate [R^2^ (0.9842–0.9972); P < 0.0001 for each triplicate]. JCAR014 CAR-T cell standard curve runs 1 and 2 were done on the same day on two different machines (the ABI QuantStudio^TM^ Real-Time PCR system and Bio-Rad CFX 384 Real-Time PCR Detection System, respectively). Run 3 was performed six days later on the same machine as run 1. (B) MAGEA1 TCR-T cell plasmid standard curves using data points from three different PCR runs, with reactions run in triplicate, on three different days [R^2^ (0.9640–0.9908); P < 0.0001 for each triplicate]. MAGEA1 TCR-T cell standard curve runs 1 and 3 were performed using the Bio-Rad CFX 384 Real-Time PCR Detection System, while run 2 was performed using the ABI QuantStudio^TM^ Real-Time PCR system.

Standard curve samples were tested multiple times to demonstrate reproducibility (Fig 1). These reactions were run in triplicate, and quantification cycle (C_q_) values were averaged. Repeat samples were run on different days and two different machines (ABI QuantStudio Real-Time PCR system and Bio-Rad CFX 384 Real Time PCR Detection System). The slope and y-intercept values, obtained from plotting the plasmid standard curves, allow for analysis using the average C_q_ values from experimental samples run in triplicate (Eq 1: $copy\;number=10^{\left(\frac{\left(Cq-intercept\right)}{\left(slope\right)}\right)}$). The number of CAR-T cells or TCR-T cells present in an experimental sample can be calculated using the average transfection numbers for each cellular product.

## Results

### Limit of detection

**Fig 2:**
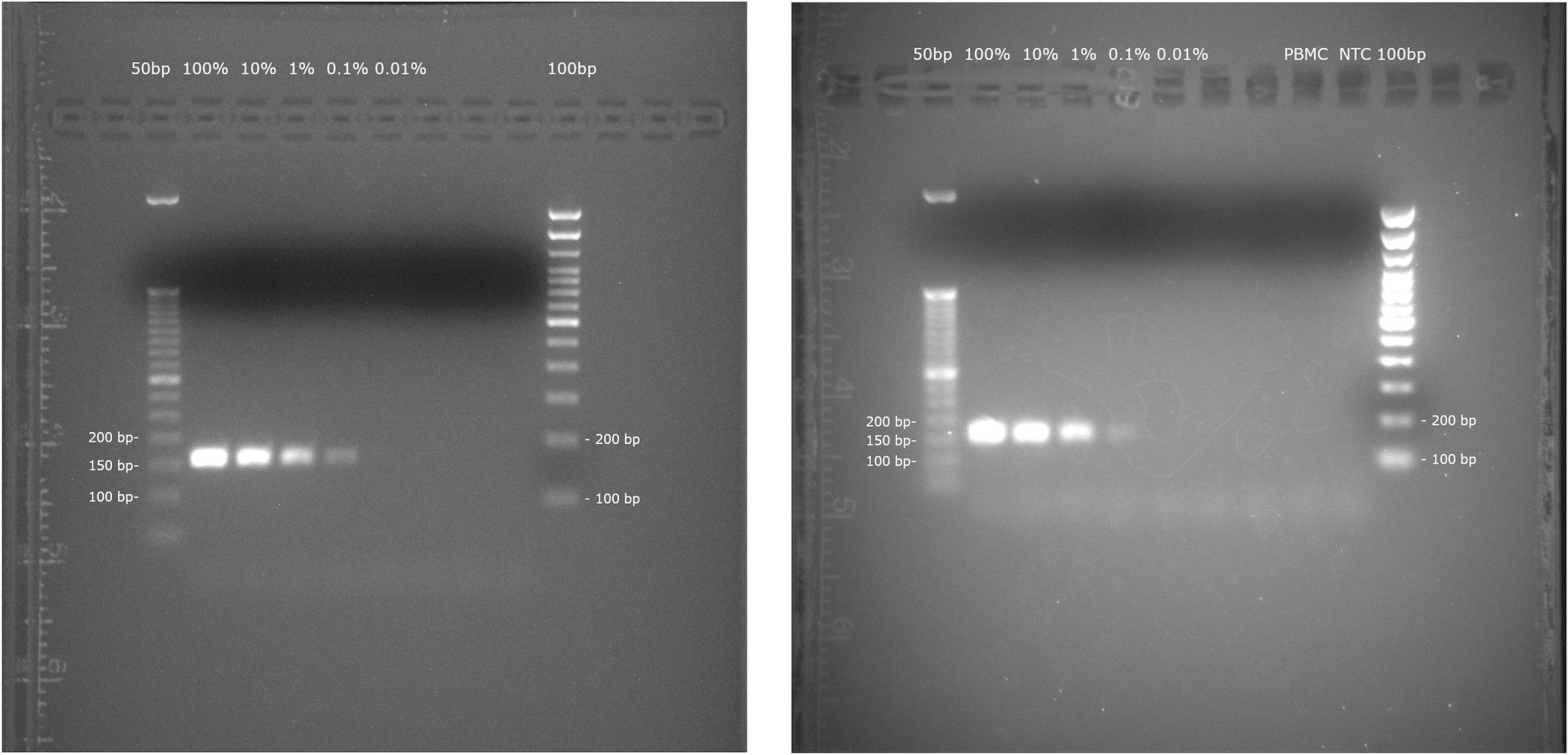
Gel electrophoresis imaging of traditional PCR products using *WPRE*- targeted primers. Left: 10-fold dilution series of fresh HA-1 TCR-T cell product DNA in a background of fresh PBMC DNA. Right: 10-fold dilution series of fresh JCAR014 CAR-T cell product DNA in a background of fresh PBMC DNA, with 100% PBMC and molecular biology grade water as negative controls. Ladders of 50 bp and 100 bp were used as size markers on 2% agarose gels shown.

To establish the limit of detection of engineered cell products, DNA extracted from CAR-T or TCR-T cells were mixed in defined weight percentages from 100% to 0.0001% with DNA extracted from normal donor PBMC cells. Traditional PCR detected DNA from adoptive cellular therapy products in dilutions down to 0.1% for JCAR014 CAR-T cells and HA-1 TCR-T cells (Fig 2). Using a SYBR Green Fluorophore qPCR assay, adoptive cellular therapy product DNA was reliably detected for CAR-T cell DNA at 0.0001%, and TCR-T cell DNA at 0.001% of fresh cell DNA composition (Table 2). These experiments and their results demonstrated the sensitivity of the assay and the specificity of the primers and parameters used to detect the *WPRE* region.

**Table 2:** SYBR green qPCR reactions. Quantification cycles (C_q_) and melt temperatures (T_m_) for serial dilutions of DNA from JCAR014 CAR-T or HA-1 TCR-T cell DNA into DNA from normal healthy donor PBMCs are shown. Sample results were considered positive if T_m_ value was between 81.00 and 84.50°C.

Sample Composition	C_q_ (avg)	T_m_ (°C)	Interpretation
100% CAR-T** **	24.48	81.00	Positive
10% CAR-T** **	29.92	81.00	Positive
1% CAR-T** **	31.19	None	Negative
0.1% CAR-T** **	32.67	81.00	Positive
0.01% CAR-T** **	36.69	81.00	Positive
0.001% CAR-T** **	34.41	81.00	Positive
0.0001% CAR-T** **	35.35	81.00	Positive
100% PBMC** **	38.78	None	Negative
100% TCR-T** **	20.37	81.00	Positive
10% TCR-T** **	25.35	81.00	Positive
1% TCR-T** **	26.40	81.00	Positive
0.1% TCR-T** **	29.49	81.00	Positive
0.01% TCR-T** **	29.69	81.00	Positive
0.001% TCR-T** **	33.75	81.00	Positive
0.0001% TCR-T** **	None	None	Negative
100% PBMC** **	38.36	None	Negative

By analyzing the T_m_ of each reaction product, and running the reaction products through gel electrophoresis, it was possible to distinguish true positive samples from false positives, which generated a C_q_ value but lacked target-specific T_m_ peaks or correctly sized bands in a gel (e.g., 100% PBMC samples in Table 2). This verification is important due to the occasional false positivity at the high cycle numbers needed for detection with low DNA inputs extracted from small archival biopsies.

### Standard curve results and reproducibility

After optimizing storage conditions, the standard curve data were overall consistent, linear, and reproducible throughout the sample testing processes for both CAR-T cell and TCR-T cell products. The standard curve data indicates the limit of detection at around 100 copies per reaction. Some qPCR reactions ran in triplicate at 100 copies of *WPRE* DNA per reaction yielded C_q_ values while others did not show any amplification.

### Validation with contrived mixtures

When we tested admixtures of engineered cell products and human PBMCs embedded together as FFPE blocks (“cell pellets” in Table 1), this assay was reliable and produced the expected results (Table 3). A contrived sample with DNA extracted from FFPE tonsil tissue spiked with CAR-T cell *WPRE* DNA was created to generate ~1000 copies per reaction and resulted in detection of 1.17E+03 copies (Table 3).

**Table 3:** qPCR results from FFPE samples using SYBR green with *WPRE*-specific primers.

Sample	Input DNA (ng)	C_q_ (avg)	*WPRE* Copy Number	*WPRE* Copy Number per ng DNA	CAR-T/TCR-T Detection Confirmed (method)
**JCAR014 CAR-T cell pellet** (0.33 CART/PBMC ratio)** **	16.56	23.45	2.21E+06	1.33E+05	Yes (ISH^a^, MCA ^b^, AGE ^c^)
**MAGEA1 TCR-T cell pellet** (0.21 TCRT/PBMC ratio)** **	48.00	24.69	2.96E+06	6.17E+04	Yes (ISH, MCA, AGE)
**TONSIL spiked with 10**^**3**^ **copies of JCAR014 plasmid/Reaction **	40.00	36.89	1.17E+03	2.93E+01	Yes (MCA, AGE)

Samples were run in triplicate in parallel with CAR-T or TCR-T plasmid DNA standard curves from which the relative *WPRE* copy number per ng of input genomic DNA was calculated.

^a^ ISH- *in situ* hybridization.

^b^ MCA- melt curve analysis.

^c^ AGE- agarose gel electrophoresis imaging.

### Detection of engineered cells in archival biopsies

The *WPRE* qPCR assay was performed on nine patients who were enrolled in unrelated clinical trials for CAR-T cell therapy at Fred Hutchinson Cancer Center. All patients’ diagnoses and treatment are presented in Table 1. Their diagnoses include non-small cell lung cancer, (n = 2, 22.2%), triple negative breast cancer (n = 5, 55.5%), diffuse large B-cell lymphoma (n = 1, 11.1%), and chronic lymphocytic leukemia (n = 1, 11.1%). One patient received JCAR014 CAR-T cells (CD19-targeted, n = 1, 11.1%), and eight patients received receptor tyrosine kinase-like orphan receptor 1 (ROR1)—targeted CAR-T cells (n = 8, 88.9%) [22]. All patient samples were collected post-CAR-T cell infusion. DNA from patient samples 1 and 6 were extracted from FFPE tissue curls while the remaining patient samples were extracted from available unstained FFPE tissue slides with varying amounts of tissue.

Four patient samples (patients 1, 2, 3, and 6, 44.4%) showed amplification during qPCR (Table 4). The remaining patient samples (55.6%) did not show amplification. Normal human tonsil tissue was used as a negative control tissue. All these samples (excluding patient 6, which had lower DNA yield) were run in triplicate. As a note, the amount of input patient DNA was maximized from these small biopsies, and therefore a variable amount of input DNA was used across the sample cohort (Table 4). All patient samples that amplified using this assay were confirmed to contain the correct product via gel electrophoresis imaging (S1 Fig) and melt curve analysis (data not shown). Regarding patient 6, the data shown are from a sample that was run in a separate experiment from the others in the cohort, as the initial biopsy selected yielded insufficient DNA. A second archival decalcified bone marrow core biopsy sample was procured for DNA extraction and analysis revealed amplification of a product. When performing melt curve analysis, peaks were seen within the correct T_m_, but did not pass the amplitude threshold as set by the Bio-Rad CFX 384 Real Time PCR Detection System instrument. However, when the PCR products were run on a 2% agarose gel, bands were seen at the appropriate size for our amplicon product in both duplicate reactions, run alongside three FFPE tonsil negative controls and three non-template controls (S1 Fig). We have interpreted this aggregate information as a positive result for patient 6. In one case (patient 1), the presence of infiltrating CAR-T cells detected by *WPRE* qPCR was corroborated by flow cytometry analysis of a paired fresh tumor biopsy at the time of tissue sampling (data not shown) and by *in situ* hybridization (ISH) staining of the same FFPE tissue biopsy [23].

**Table 4:** List of real-time PCR results from patient FFPE samples using SYBR green qPCR with *WPRE*-specific primers.

Sample	Cell Target	Amount of Input DNA (ng)	C_q_ (avg)	*WPRE* Copy Number	*WPRE* Copy Number per ng	CAR-T/TCR-T Detected? (method)
**Patient 1 **	CD19	24.10	39.37	1.68E+02	6.97E+00	Yes (ISH, FC^d^, MCA, AGE)
**Patient 2 **	ROR1	13.20	34.62	1.23E+03	9.30E+01	Yes (MCA, AGE)
**Patient 3 **	ROR1	3.98	35.02	9.52E+02	2.39E+02	Yes (MCA, AGE)
**Patient 4 **	ROR1	132.00	-	0	0	No (MCA, AGE)
**Patient 5 **	ROR1	9.52	-	0	0	No (MCA, AGE)
**Patient 6 **	ROR1	7.12	38.04	1.89E+01	2.66E+00	Yes (MCA, AGE)
**Patient 7 **	ROR1	18.80	-	0	0	No (MCA, AGE)
**Patient 8 **	ROR1	0.66	-	0	0	No (MCA, AGE)
**Patient 9 **	ROR1	7.02	-	0	0	No (MCA, AGE)
**Healthy donor tonsil**	N/A^e^	178.00	-	0	0	No (MCA, AGE)
**Non-template control**	N/A	0	-	0	0	No (MCA, AGE)

Patient samples copy number calculations were done based on standard curves of plasmid DNA suspended in human tonsil FFPE DNA background. A variable amount of input DNA was used across the cohort to maximize sensitivity.

^d^ FC- flow cytometry.

^e^ N/A- not applicable.

### Range of detection

This assay detected *WPRE* DNA in fresh, frozen, or FFPE tissues. Standard curve analysis defined a limit of detection of 100 copies per reaction. We noted a loss of reaction stability with DNA inputs greater than 230 ng. With such high template concentrations, the C_q_ values were too low to calculate copy numbers using the plasmid standard curves and pre-amplification or non-specific peaks were frequently observed. The lowest input DNA value tested from a patient biopsy where *WPRE* DNA was detected was 3.98 ng. One sample was tested at a DNA input value lower than this, with 0.66 ng per reaction, which yielded a negative result.

### Orthogonal confirmation

The presence or absence of *WPRE* signal we observed was as expected for the patient samples based on the clinical history and timepoint of treatment with adoptive cellular therapy products. Additionally, the FFPE biopsy sample from patient 1 was selected for our assay validation based on confirmation in prior studies to have had CAR-T cells present using *WPRE* ISH [23]. This allowed for a comparison of our results with previously published imaging, to serve as orthogonal confirmation that this biopsy did contain infiltrating CAR-T cells. Using this confirmation as a guideline, we were able to verify detection in the other experimental samples based on matching melt curve analysis and gel imaging.

## Discussion

The ability to detect CAR-T/TCR-T cells in archival tissue as opposed to fresh tissue is important, as most clinical pathology workflows are centered around FFPE tissues which provide the best morphology for pathological review and diagnosis. Extracting DNA from FFPE tissue has been historically challenging, as nucleic acids tend to fragment when subjected to formalin fixation and resultant DNA cross-linkage [24]. We designed our PCR amplicon to be small, less than 160 bp, which is the typical minimum fragment length associated with formalin fragmentation. Additionally, we devised our experiments using both fresh and FFPE preserved samples made from the same cell lines and tissues when available to show the capability to detect the CAR-T and TCR-T products in FFPE tissues. Although this assay was developed for archival FFPE tumor biopsies, future application could include prospective samples such fine needle aspirations for monitoring of adoptive cellular therapy progress.

We developed the standard curves to determine our limit of detection and for use in quantifying *WPRE* copy number in patient samples. Our assay could detect less than or equal to 100 *WPRE* copies per reaction reliably. We validated and tested the assay on a variety of FFPE samples, including small needle biopsies with low DNA yield, and demonstrated the capability to detect CAR-T and TCR-T cells in these tissues. When accounting for the average transfection copy number in many of our products (1–5 *WPRE* copies per cell), detecting 100 copies equates to the ability to detect as few as 20 cells per sample. However, this level of sensitivity may still be too low for detection in small FFPE needle core biopsies that have limited T cell infiltrate. Another limitation of this assay as currently configured is the need for a robust and reproducible standard curve to measure copy number in a patient sample. Generation of CAR-T/TCR-T cell DNA standard curves is time consuming and requires careful validation as well as access to pre-infusion product or vector DNA. Plans to convert this assay to a digital PCR format will obviate the need for a standard curve and streamline the workflow. Moreover, a digital PCR format may further increase the assay’s sensitivity.

Four out of the nine patient samples tested with this assay yielded positive results for *WPRE* amplification, and two of the positive samples were small needle biopsies with limited material. This sensitive detection is important in that small needle biopsies are usually all that is available for test in a histology/pathology lab. The remaining patient samples did not amplify *WPRE* DNA when tested. This may be due to a lack of CAR-T cells present in the tumor, rendering these as true negatives. However, the DNA input from these FFPE samples may also have been too low to show amplification if the amount of CAR-T cell infiltration was low.

There are several assays used to detect CAR-T/TCR-T cells in tissue reported in previous literature, which include detection by flow cytometry, ISH, and immunohistochemistry (IHC) [2,23,25–28]. Flow cytometric analysis allows for the identification and characterization of the CAR-T/TCR-T cell subpopulation and the patient’s own immune cells using peripheral blood samples. However, flow cytometry assays require fresh or frozen viable cells and thus are time sensitive. Tumor biopsies are generally processed as FFPE samples for diagnosis and archiving, at which point IHC, which is routinely performed on FFPE tissue sections, can be used to detect T-cells with antibodies against CD3, CD4, and CD8. However, this technique does not distinguish CAR-T/TCR-T cells from endogenous T-cells. Antibodies specific for detection of CAR-T cells in IHC have been reported, but their sensitivity is unknown, and they are only useful for specific CAR-T constructs [29]. Prior studies using *WPRE* RNA ISH and unique CAR-specific sequences have also been reported by us and others [23,28]. The RNA-based *WPRE* ISH assay previously reported by our group, although broadly applicable and useful for providing spatial information, is inherently less reliable due to the lability of RNA. Although lacking the spatial context of other methods, this DNA-based PCR detection assay is more reliable as DNA is more stable than RNA in archival samples. Previous qPCR assays were developed to measure the *WPRE* copy number in DNA extracted from fresh lentiviral transduced cells, but these other assays were not optimized for use on FFPE samples [30].

This assay has potential utility in both research and clinical applications. In research contexts, this assay can be used in ongoing translational research studies to investigate CAR-T cell and TCR-T cell trafficking. Insight into whether engineered cells are homing, expanding, and persisting in tumor tissues can inform researchers of immunotherapy efficacy and barriers to efficacy. This provides a more complete picture of how engineered T cells operate in the body and is crucial in the development of more effective immunotherapies. In clinical settings, this assay can be used to test serial samples from baseline and through the course of treatment to understand tumor site trafficking as one variable contributing to the effectiveness of immunotherapy. This assay provides an avenue for retrospective analysis of past patient cases to assess product infiltration into tumors. Analysis using our assay on patient FFPE samples provided insight as to how these cells disseminated and persisted *in vivo*.

The risks of developing CRS and ICANS are major concerns with CAR-T/TCR-T cell therapy. Additional AEs such as macrophage activation syndrome / hemophagocytic lymphohistiocytosis have also occurred as variants of CRS requiring different treatment approaches [31]. Furthermore, interstitial pneumonitis not involving CRS has recently been reported, as have cutaneous toxicities [32,33]. Assaying *WPRE* in tissue biopsies from sites of AEs via qPCR could contextualize engineered T cells’ contributions to tissue specific AEs, which can inform clinical decision making in turn. As current understandings of immunotherapies’ side effects are evolving, this assay provides useful insight into cellular trafficking. As adoptive cellular therapies grow more commonplace and further applications for them arise, the need for this assay and others like it will also increase. Many current cellular products are specifically restricted to their targets. However, as new, less restricted products emerge, it is important to monitor whether resulting side effects and second-site toxicities are correlated with CAR-T/TCR-T cell presence at those sites.

We propose a simple, novel, and cost-effective assay with a rapid turnaround time and broad sample type applicability for detecting tissue-specific CAR-T/TCR-T cell presence that is sensitive, specific, and applicable to different cell products. Our assay could allow clinicians and researchers to monitor the trafficking of these cells throughout the body, to tumors, as well as diagnose tissue-specific AEs, which opens a new line of diagnostics. We plan to continue testing this assay on tissue samples obtained from patients who were treated with approved, commercially available immunotherapy products. Given the common use of *WPRE* enhancer sequences in lentiviral vectors, this assay is likely to be even more broadly applicable [34–36].

## Supporting information

S1 Fig ROR1 CAR-T patient sample 2% agarose gel. Patient numbers are labeled. Tonsil negative control is indicated by the letter “T”. NTC used was molecular biology grade water. The qPCR products shown in images (a) and (b) were run on the same day. The qPCR reaction using FFPE DNA from patient 6 shown on image (c) was run in duplicate on a different day, as the initial sample for this patient did not yield sufficient DNA.
(TIF)

S1 Raw images (PDF)
