# Supplementary figures and images for: qPCR assay for detection of Woodchuck Hepatitis Virus Post-Transcriptional Regulatory Elements from CAR-T and TCR-T cells in fresh and formalin-fixed tissue
Source: PLoS One. 2024 Jun 6;19(6):e0303057. doi: 10.1371/journal.pone.0303057 (PMC11156344; doi:10.1371/journal.pone.0303057)

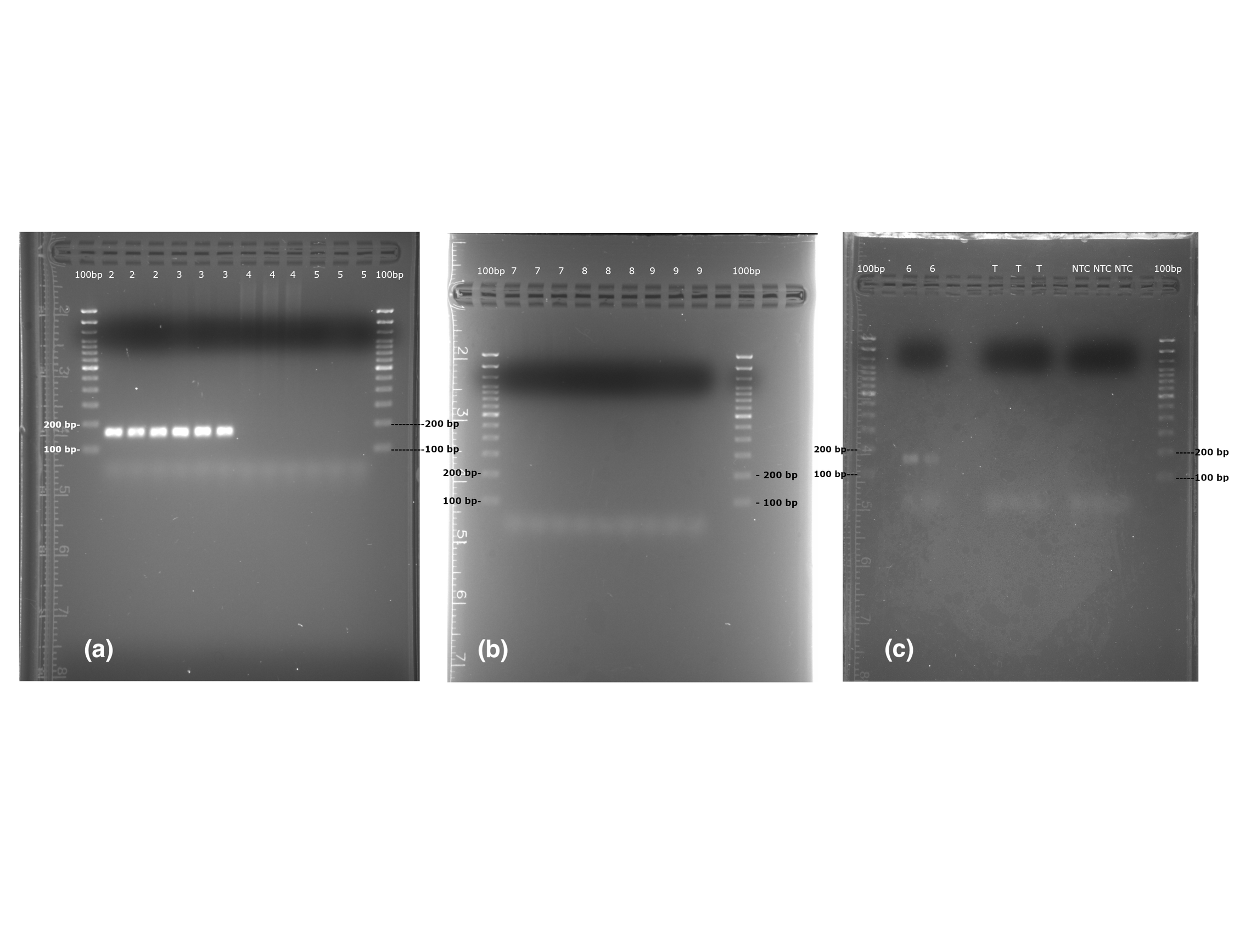

Supplement: S1 Fig — Patient numbers are labeled. Tonsil negative control is indicated by the letter “T”. NTC used was molecular biology grade water. The qPCR products shown in images (a) and (b) were run on the same day. The qPCR reaction using FFPE DNA from patient 6 shown on image (c) was run in duplicate on a different day, as the initial sample for this patient did not yield sufficient DNA. (TIF) [file pone.0303057.s001.tif]
