# Supplementary material for: qPCR assay for detection of Woodchuck Hepatitis Virus Post-Transcriptional Regulatory Elements from CAR-T and TCR-T cells in fresh and formalin-fixed tissue
Source: PLoS One. 2024 Jun 6;19(6):e0303057. doi: 10.1371/journal.pone.0303057 (PMC11156344; doi:10.1371/journal.pone.0303057)
Supplement: S1 Raw images — (PDF) [file pone.0303057.s002.pdf]

Fig1 (Left)- HA-1 TCR-T Dilution Series
Loading order (left-to-right): blank, blank, 50bp ladder, 100%, 10%, 1%, 0.1%, 0.01%, blank, blank, blank, 100bp ladder, blank, blank, blank
Image captured using BioDoc-It2 Imager and UVP Software

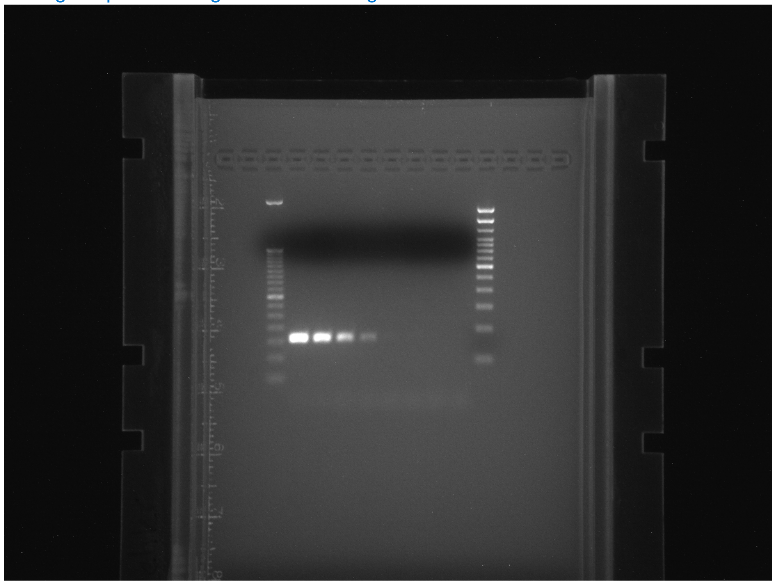

Fig1 (Right)- JCAR014 CAR-T Dilution Series
Loading order (left-to-right): blank, blank, 50bp ladder, 100%, 10%, 1%, 0.1%, 0.01%, blank, blank, PBMC, NTC, 100bp ladder, blank, blank
Image captured using BioDoc-It2 Imager and UVP Software

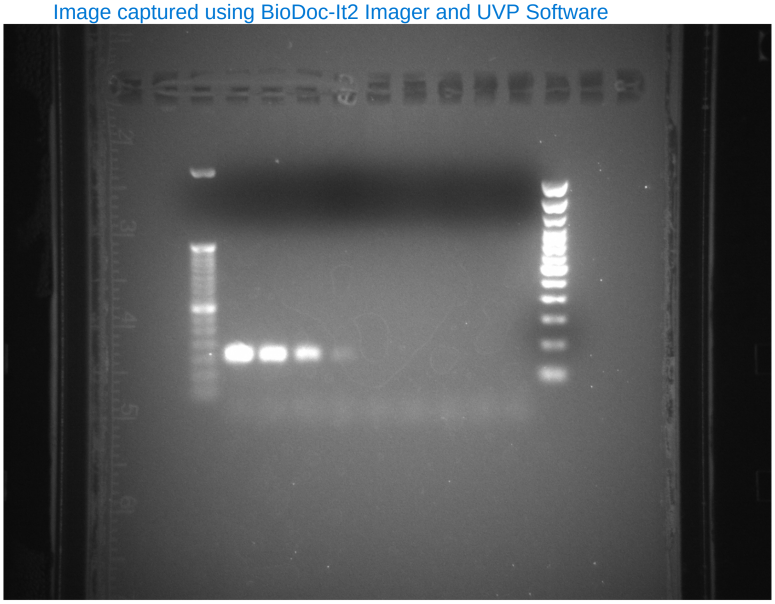

S1 Fig (a) Patient Samples.
Loading order (left-to-right): blank, 100bp ladder, 2, 2, 2, 3, 3, 3, 4, 4, 4, 5, 5, 5, 100bp ladder
Image captured using BioDoc-It2 Imager and UVP Software

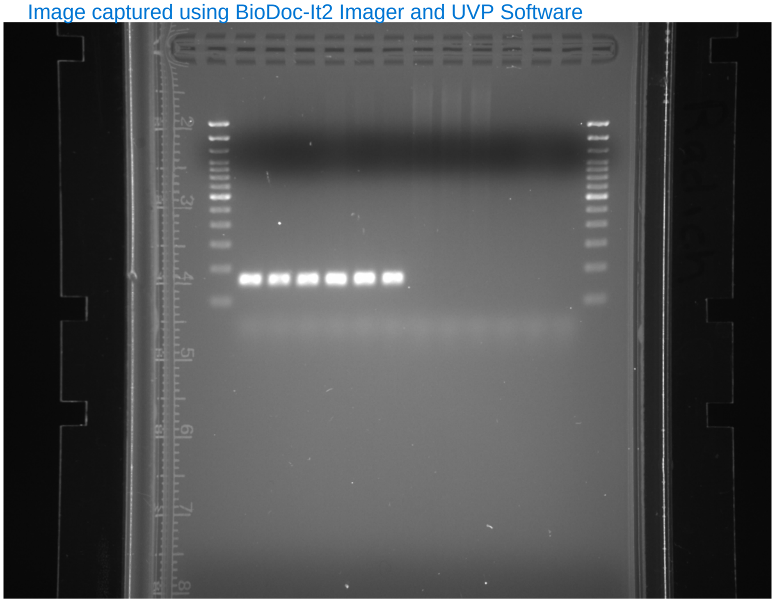

S1 Fig (b) Patient Samples.
Loading order (left-to-right): blank, 100bp ladder, 7, 7, 7, 8, 8, 8, 9, 9, 9, blank, 100bp ladder, blank, blank
Image captured using BioDoc-It2 Imager and UVP Software

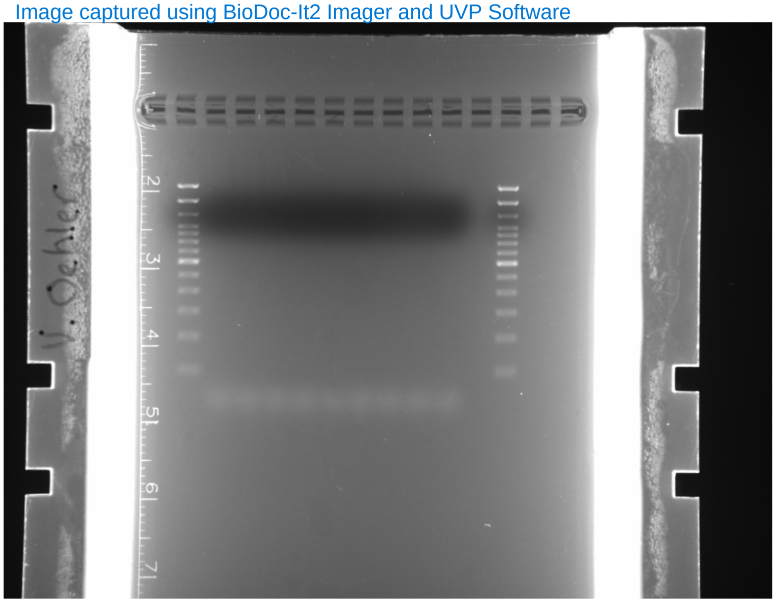

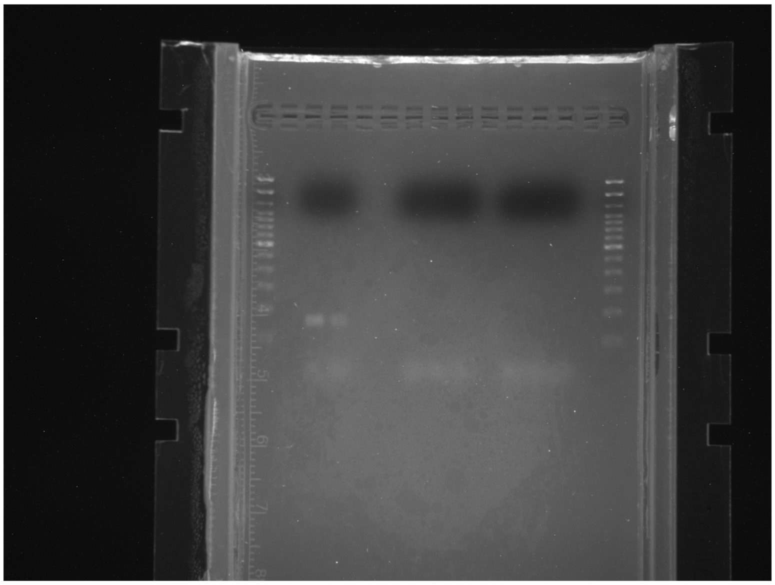

S1 Fig (c) Patient Samples
Loading Order (left-to-right): 100bp ladder, blank, 6, 6, blank, blank, tonsil, tonsil, tonsil, blank, NTC, NTC, NTC, blank, 100bp ladder

Image captured using BioDoc-It2 Imager and UVP Software
